# Supplementary material for: End Sequence Analysis Toolkit (ESAT) expands the extractable information from single-cell RNA-seq data
Source: Genome Res. 2016 Oct;26(10):1397–410. doi: 10.1101/gr.207902.116 (PMC5052061; doi:10.1101/gr.207902.116)
Supplement: Supplemental Material [file supp_gr.207902.116_Supp_Materials.tar.gz › ESAT-esat_v0.1/doc/html/classbroad_1_1pda_1_1seq_1_1graph_1_1_chromosome_with_bubbles_j_graph_t-members.html]

ESAT: Member List


ESAT


- Main Page
- Related Pages
- Packages
- Classes
- Files

- Class List
- Class Index
- Class Hierarchy
- Class Members

All Classes Namespaces Files Functions Variables Enumerator Pages

- broad
- pda
- seq
- graph
- ChromosomeWithBubblesJGraphT

broad.pda.seq.graph.ChromosomeWithBubblesJGraphT Member List

This is the complete list of members for broad.pda.seq.graph.ChromosomeWithBubblesJGraphT, including all inherited members.

|  |  |  |
| --- | --- | --- |
| addEdge(Annotation v1, Annotation v2, BubbleEdge e) | broad.pda.seq.graph.ChromosomeWithBubblesJGraphT |  |
| addEdgeAddingMissingVertices(Annotation intron, float count) | broad.pda.seq.graph.ChromosomeWithBubblesJGraphT |  |
| addNewEdge(BubbleEdge e, Annotation v1, Annotation v2) | broad.pda.seq.graph.ChromosomeWithBubblesJGraphT |  |
| addVertex(Annotation v) | broad.pda.seq.graph.ChromosomeWithBubblesJGraphT |  |
| checkOrientation(Collection< BubbleEdge > edges) | broad.pda.seq.graph.ChromosomeWithBubblesJGraphT |  |
| ChromosomeWithBubblesJGraphT(String name) | broad.pda.seq.graph.ChromosomeWithBubblesJGraphT |  |
| ChromosomeWithBubblesJGraphT(String name, int start, int end) | broad.pda.seq.graph.ChromosomeWithBubblesJGraphT |  |
| ChromosomeWithBubblesJGraphT(String name, Collection<?extends Annotation > vertices, Collection<?extends Annotation > edges, Map<?extends Annotation, Double > edgeCounts, Map<?extends Annotation, Double > nodeCounts, double lambda, double numMarkers, double numReads, int minSplices) | broad.pda.seq.graph.ChromosomeWithBubblesJGraphT |  |
| ChromosomeWithBubblesJGraphT(String name, Collection<?extends Annotation > vertices, Collection<?extends Annotation > edges) | broad.pda.seq.graph.ChromosomeWithBubblesJGraphT |  |
| ChromosomeWithBubblesJGraphT(String name, Map<?extends Annotation, Double > nodes, Map< BubbleEdge, Double > edges, double lambda, double numMarkers, double numReads, double minEdgeSupport) | broad.pda.seq.graph.ChromosomeWithBubblesJGraphT |  |
| ChromosomeWithBubblesJGraphT(String name, Collection<?extends Annotation > nodes, Collection<?extends Annotation > edges, double lambda, double numMarkers, double numReads, int minSplices) | broad.pda.seq.graph.ChromosomeWithBubblesJGraphT |  |
| createEdge(Alignments connection, Annotation v1, Annotation v2, Integer count, EdgeSourceType type) | broad.pda.seq.graph.ChromosomeWithBubblesJGraphT |  |
| findEdge(int start, int end) | broad.pda.seq.graph.ChromosomeWithBubblesJGraphT |  |
| findReachableTargetVertices(Annotation source, Collection< Annotation > alreadyTraversed, Collection< Annotation > sinkVertices, Collection< Annotation > reachableSinks, int counter, DirectedGraph< Annotation, BubbleEdge > g) | broad.pda.seq.graph.ChromosomeWithBubblesJGraphT |  |
| getAllPaths() | broad.pda.seq.graph.ChromosomeWithBubblesJGraphT |  |
| getAllPaths(double minSpliceFrequency) | broad.pda.seq.graph.ChromosomeWithBubblesJGraphT |  |
| getBackwardEdges(Annotation vertex, double alpha, Collection< EdgeSourceType > validTypes) | broad.pda.seq.graph.ChromosomeWithBubblesJGraphT |  |
| getBackwardPaths(int start, int end, double alpha, Collection< EdgeSourceType > validTypes) | broad.pda.seq.graph.ChromosomeWithBubblesJGraphT |  |
| getChromosomeLength() | broad.pda.seq.graph.ChromosomeWithBubblesJGraphT |  |
| getEdges(Collection< Annotation > vertices) | broad.pda.seq.graph.ChromosomeWithBubblesJGraphT |  |
| getEnd() | broad.pda.seq.graph.ChromosomeWithBubblesJGraphT |  |
| getForwardEdges(Annotation vertex, double alpha, Collection< EdgeSourceType > validTypes) | broad.pda.seq.graph.ChromosomeWithBubblesJGraphT |  |
| getGenePaths(double alpha) | broad.pda.seq.graph.ChromosomeWithBubblesJGraphT |  |
| getGenePaths(double alpha, double minSpliceFrequency) | broad.pda.seq.graph.ChromosomeWithBubblesJGraphT |  |
| getIncidentEdges(Annotation vertex) | broad.pda.seq.graph.ChromosomeWithBubblesJGraphT |  |
| getInOrUnorientedEdges(Annotation v) | broad.pda.seq.graph.ChromosomeWithBubblesJGraphT |  |
| getLambda() | broad.pda.seq.graph.ChromosomeWithBubblesJGraphT |  |
| getLocalRate(Annotation exon) | broad.pda.seq.graph.ChromosomeWithBubblesJGraphT |  |
| getLocalRate() | broad.pda.seq.graph.ChromosomeWithBubblesJGraphT |  |
| getName() | broad.pda.seq.graph.ChromosomeWithBubblesJGraphT |  |
| getNodePair(BubbleEdge edge) | broad.pda.seq.graph.ChromosomeWithBubblesJGraphT |  |
| getNumberOfMarkers() | broad.pda.seq.graph.ChromosomeWithBubblesJGraphT |  |
| getNumberOfReads() | broad.pda.seq.graph.ChromosomeWithBubblesJGraphT |  |
| getOppositeVertex(Annotation v, BubbleEdge e) | broad.pda.seq.graph.ChromosomeWithBubblesJGraphT |  |
| getOrphanNodes() | broad.pda.seq.graph.ChromosomeWithBubblesJGraphT |  |
| getOrphanNodes(Alignments region) | broad.pda.seq.graph.ChromosomeWithBubblesJGraphT |  |
| getOrphanPaths() | broad.pda.seq.graph.ChromosomeWithBubblesJGraphT |  |
| getOutOrUnorientedEdges(Annotation v) | broad.pda.seq.graph.ChromosomeWithBubblesJGraphT |  |
| getOverlappingVertices(int start, int end) | broad.pda.seq.graph.ChromosomeWithBubblesJGraphT |  |
| getPaths(double alpha) | broad.pda.seq.graph.ChromosomeWithBubblesJGraphT |  |
| getPaths(double alpha, double minSpliceFrequency) | broad.pda.seq.graph.ChromosomeWithBubblesJGraphT |  |
| getPaths(Annotation startNode, Annotation endNode, double alpha, double minSpliceFreq, DirectedGraph< Annotation, BubbleEdge > g) | broad.pda.seq.graph.ChromosomeWithBubblesJGraphT |  |
| getPaths(Annotation startNode, Annotation endNode, double alpha, Collection< EdgeSourceType > validEdgeTypes, double minSpliceFreq, DirectedGraph< Annotation, BubbleEdge > g) | broad.pda.seq.graph.ChromosomeWithBubblesJGraphT |  |
| getPaths(int start, int end) | broad.pda.seq.graph.ChromosomeWithBubblesJGraphT |  |
| getPaths(int start, int end, boolean forward) | broad.pda.seq.graph.ChromosomeWithBubblesJGraphT |  |
| getPaths(int start, int end, double alpha, boolean forward) | broad.pda.seq.graph.ChromosomeWithBubblesJGraphT |  |
| getPaths(int start, int end, double alpha, Collection< EdgeSourceType > validTypes) | broad.pda.seq.graph.ChromosomeWithBubblesJGraphT |  |
| getRelativeGenomeLength() | broad.pda.seq.graph.ChromosomeWithBubblesJGraphT |  |
| getRPKMConstant() | broad.pda.seq.graph.ChromosomeWithBubblesJGraphT |  |
| getSinkVertices(Collection< EdgeSourceType > validTypes, DirectedGraph< Annotation, BubbleEdge > g) | broad.pda.seq.graph.ChromosomeWithBubblesJGraphT |  |
| getSourceVertices(Collection< EdgeSourceType > validTypes, DirectedGraph< Annotation, BubbleEdge > g) | broad.pda.seq.graph.ChromosomeWithBubblesJGraphT |  |
| getSpliceWeight() | broad.pda.seq.graph.ChromosomeWithBubblesJGraphT |  |
| getStart() | broad.pda.seq.graph.ChromosomeWithBubblesJGraphT |  |
| getValidOutgoingEdges(Annotation vertex, double alpha, Collection< EdgeSourceType > validTypes, DirectedGraph< Annotation, BubbleEdge > g) | broad.pda.seq.graph.ChromosomeWithBubblesJGraphT |  |
| inOrientedDegree(Annotation v, Collection< EdgeSourceType > validTypes, DirectedGraph< Annotation, BubbleEdge > g) | broad.pda.seq.graph.ChromosomeWithBubblesJGraphT |  |
| isGap(int start, int end) | broad.pda.seq.graph.ChromosomeWithBubblesJGraphT |  |
| isInAllGaps(int start, int end) | broad.pda.seq.graph.ChromosomeWithBubblesJGraphT |  |
| isSpanningGap(int start, int end) | broad.pda.seq.graph.ChromosomeWithBubblesJGraphT |  |
| iterator(int windowSize, int start, int overlap) | broad.pda.seq.graph.ChromosomeWithBubblesJGraphT |  |
| iterator(int windowSize, int start, int overlap, boolean skipGapSpanningWindows) | broad.pda.seq.graph.ChromosomeWithBubblesJGraphT |  |
| iterator(int windowSize, int start) | broad.pda.seq.graph.ChromosomeWithBubblesJGraphT |  |
| outOrientedDegree(Annotation v, Collection< EdgeSourceType > validTypes, DirectedGraph< Annotation, BubbleEdge > g) | broad.pda.seq.graph.ChromosomeWithBubblesJGraphT |  |
| removeEdge(BubbleEdge e) | broad.pda.seq.graph.ChromosomeWithBubblesJGraphT |  |
| removeSpanningGapNodes() | broad.pda.seq.graph.ChromosomeWithBubblesJGraphT |  |
| removeVertex(Annotation vertex) | broad.pda.seq.graph.ChromosomeWithBubblesJGraphT |  |
| setChromosomeLength(int chromosomeLength) | broad.pda.seq.graph.ChromosomeWithBubblesJGraphT |  |
| setEnd(int end) | broad.pda.seq.graph.ChromosomeWithBubblesJGraphT |  |
| setExonScores(Annotation node, Double score) | broad.pda.seq.graph.ChromosomeWithBubblesJGraphT |  |
| setLocalRate(Collection< Path > paths) | broad.pda.seq.graph.ChromosomeWithBubblesJGraphT |  |
| setLocalRate(Map< Annotation, Double > localRateMap) | broad.pda.seq.graph.ChromosomeWithBubblesJGraphT |  |
| setRPKMConstant(double num) | broad.pda.seq.graph.ChromosomeWithBubblesJGraphT |  |
| setSpliceWeight(double spliceWeight) | broad.pda.seq.graph.ChromosomeWithBubblesJGraphT |  |
| setStart(int start) | broad.pda.seq.graph.ChromosomeWithBubblesJGraphT |  |
| writeGraph(String save, boolean setUpShortExonLabels) | broad.pda.seq.graph.ChromosomeWithBubblesJGraphT |  |
| writeGraph(String save) | broad.pda.seq.graph.ChromosomeWithBubblesJGraphT |  |
| writeGraph(String save, Alignments region) | broad.pda.seq.graph.ChromosomeWithBubblesJGraphT |  |
| writeGraph(String save, Alignments region, boolean setUpShortExonLabels) | broad.pda.seq.graph.ChromosomeWithBubblesJGraphT |  |
| writeGraph(String save, Alignments region, boolean setUpShortExonLabels, String graphName) | broad.pda.seq.graph.ChromosomeWithBubblesJGraphT |  |


---

Generated on Fri Aug 15 2014 15:11:02 for ESAT by  

 1.8.7
